# Neurologic Abnormalities in Workers of a 1-Bromopropane Factory

**DOI:** 10.1289/ehp.6995

**Published:** 2004-06-30

**Authors:** Gaku Ichihara, Weihua Li, Eiji Shibata, Xuncheng Ding, Hailan Wang, Yideng Liang, Simeng Peng, Seiichiro Itohara, Michihiro Kamijima, Qiyuan Fan, Yunhui Zhang, Enhong Zhong, Xiaoyun Wu, William M. Valentine, Yasuhiro Takeuchi

**Affiliations:** ^1^Field of Social Life Science, Nagoya University Graduate School of Medicine, Nagoya, Japan; ^2^Shanghai Institute of Planned Parenthood Research, Shanghai, China; ^3^Department of Health and Psychosocial Medicine, Aichi Medical University, Aichi, Japan; ^4^Division of Neurobiology, Department of Psychiatry, Johns Hopkins University School of Medicine, Baltimore, Maryland, USA; ^5^Yixing Anti-Epidemic and Health Station, Yixing, China; ^6^Department of Pathology, Vanderbilt University Medical Center, Nashville, Tennessee, USA; ^7^Emeritus Professor, Nagoya University, Nagoya, Japan

**Keywords:** 1-bromopropane, distal latency, nerve conduction velocity, neurobehavioral testing, neurotoxicity, ozone-depleting solvents, postural sway testing, reproductive toxicity, vibration sense

## Abstract

We reported recently that 1-bromopropane (1-BP; *n*-propylbromide, CAS Registry no. 106-94-5), an alternative to ozone-depleting solvents, is neurotoxic and exhibits reproductive toxicity in rats. The four most recent case reports suggested possible neurotoxicity of 1-BP in workers. The aim of the present study was to establish the neurologic effects of 1-BP in workers and examine the relationship with exposure levels. We surveyed 27 female workers in a 1-BP production factory and compared 23 of them with 23 age-matched workers in a beer factory as controls. The workers were interviewed and examined by neurologic, electrophysiologic, hematologic, biochemical, neurobehavioral, and postural sway tests. 1-BP exposure levels were estimated with passive samplers. Tests with a tuning fork showed diminished vibration sensation of the foot in 15 workers exposed to 1-BP but in none of the controls. 1-BP factory workers showed significantly longer distal latency in the tibial nerve than did the controls but no significant changes in motor nerve conduction velocity. Workers also displayed lower values in sensory nerve conduction velocity in the sural nerve, backward recalled digits, Benton visual memory test scores, pursuit aiming test scores, and five items of the Profile of Mood States (POMS) test (tension, depression, anxiety, fatigue, and confusion) compared with controls matched for age and education. Workers hired after May 1999, who were exposed to 1-BP only (workers hired before 1999 could have also been exposed to 2-BP), showed similar changes in vibration sense, distal latency, Benton test scores, and depression and fatigue in the POMS test. Time-weighted average exposure levels in the workers were 0.34–49.19 ppm. Exposure to 1-BP could adversely affect peripheral nerves or/and the central nervous system.

Ozone-depleting solvents, such as specific chlorofluorocarbons and 1,1,1-trichloroethane, have been banned since 1996 in developed countries. Because they were used in large amounts in various industries, alternative compounds were introduced to the workplace. One such alternative compound is 1-bromopropane (1-BP; *n*-propylbromide, CAS Registry no. 106-94-5), which is used in the United States and Japan as a cleaning agent for metals, precision instruments, electronics, optical instruments, and ceramics (Ichihara, in press). It is also used in spray form as an adhesive in the United States (Ichihara et al. 2002). Environ Tech (2001) estimated the total amount of 1-BP commercially available for sale in the United States in the year 2000 was 1,967.9 metric tons (4,338,583 lb), which is comparable to 9.0, 31.0, and 10.6% of the amount of methylene chloride, perchloroethylene, and trichloroethylene used in adhesive/foam fabrication and metal cleaning in the same year in the United States. In Japan, the amount of 1-BP sold in 2003 was 1,125 metric tons, which is about double the 645 metric tons sold in 1998 (Association of Bromopropane Producers of Japan, unpublished data). In addition, in the workplace where cases of neurotoxicity had been reported, 1-BP was introduced as an alternative for methylene chloride (Ichihara et al. 2002). The benefits of using 1-BP instead of the chlorinated carbons are not clear. However, under pressure to regulate the use of chlorocarbons, 1-BP has been used as a surrogate, which is encouraged by the lack of measures to define the exposure limits. In this regard, previous animal studies revealed neurotoxicity and reproductive toxicity of 1-BP (Ichihara et al. 2000a, 2000b; Wang et al. 2002, 2003; Yamada et al. 2003; Yu et al. 1998, 2001). Exposure to 1-BP resulted in a dose-dependent limb muscle weakness and reduction of nerve conduction in rats (Ichihara et al. 2000a). It also resulted in myelin degeneration of peripheral nerves and swelling of preterminal axons in the medulla oblongata (Ichihara et al. 2000a). It was also revealed that 1-BP exhibits reproductive toxicity in both male and female rats (Ichihara et al. 2000b; Yamada et al. 2003). Thus, animal studies preceded human studies and warned about the potential neurotoxicity and reproductive toxicity of 1-BP in humans. The most recently reported cases also confirmed the neurotoxicity of 1-BP in humans (Ichihara et al. 2002; Sclar 1999). However, these case reports have limitations in terms of quantitative analysis. In 1999 we investigated a 1-BP factory, but this investigation was also limited because it was originally oriented to study the effects of 2-bromopropane (2-BP), which targets mainly reproductive and hematopoietic systems (Ichihara et al. 2004).

The aim of the present study was to assess the neurologic function and other health-related changes in workers exposed to 1-BP and compare the results with those of control workers in a beer factory.

## Materials and Methods

### Factories and workers.

The subjects were female workers of a 1-BP production factory located in Yixing, Jiangsu Province, China. The survey was conducted 16–18 January 2001. The same factory mainly produced 2-BP in 1996 (Ichihara et al. 1999), but shifted the main production to 1-BP between 1996 and 1999 (Ichihara et al. 2004), and the product was only 1-BP at the time of the present survey. 1-BP was synthesized by incubating *n*-propranolol and hydrogen bromide under concentrated sulfuric acid. The product was purified by distillation and temporarily stored in ceramic containers. The crude product was then transferred to 20-L plastic vessels through hose pipe from the cock of the container and subsequently neutralized with hydrogen carbonate. The product was finally transferred to 1,000-L drums for storage and transport. The workers were at risk of exposure to 1-BP when *a*) placing the chemicals into the reaction pots; *b*) sitting close to the reaction pots to observe and record the temperature; *c*) taking out the crude product; *d*) adding the hydrogen carbonate and stirring; and *e*) pouring the product into the drums. In the final step, the workers added the product with hand scoops to adjust the product volume in the drum.

The surveyed factory has two similar-sized manufacturing plants, each measuring 9.7 × 24.4 × 7 m (width × depth × height). In each plant, a ventilating fan was ineffectively installed 6 m from the floor; no local ventilation fan was installed in the vicinity of the areas where workers might be exposed to 1-BP. The 27 surveyed workers who were engaged in the production of 1-BP in the factory were all female. As controls, we selected age-matched (± 2 years) females at random from 202 female workers in a beer factory in the same city. The control workers lived in the same area.

In the analysis of paired *t*-tests between 1-BP workers and controls, four 1-BP workers were excluded because no corresponding match of control workers from the beer factory could be recruited. However, the analysis by exposure level or period of exposure included those 1-BP female workers for whom no corresponding age-matched controls could be recruited. All workers who were hired after 1991 and for whom corresponding age-matched controls could be recruited were identified as 1991 workers. Among them, the workers who were hired after 1999 and were exposed only to 1-BP were defined as 1999 workers.

### Medical examination.

Signed informed consent was obtained from each worker for all examinations and interviews, according to the Declaration of Helsinki (World Medical Association 2002). All female workers in the 1-BP factory and the 23 age-matched beer-factory workers were clinically examined by a trained Chinese neurologist who was conducting medical research at the Department of Neurology, Nagoya University, Japan, and had a good command of both Chinese and Japanese languages.

The vibration sensation was evaluated using a vibrating tuning fork (128 Hz); the fork was placed on the dorsum of the metatarsophalangeal joint of the big toe or the dorsum of the metacarpophalangeal joint of the thumb, and the worker was asked to report when the vibration ceased. Immediately after reporting, the tuning fork was moved to the same site (big toe or thumb) of the examiner and the duration of the lasting vibration after the worker’s report was recorded. It was difficult to assess the actual time when the delay time was < 2 sec, because it took some time (but < 2 sec) to move the tuning fork from the worker’s body to the examiner’s body. In addition, one worker reported total loss of vibration sense in the right toe. Therefore, the value could not be treated as a continuous value in the statistical analysis. The examiner was a trained female (38-year-old) neurologist who worked with every worker throughout the investigation.

### Electrophysiologic studies.

We conducted electrophysiologic studies in an air-conditioned room maintained at 24°C. The workers were acclimated to the room temperature for 30 min before the electrophysiologic studies. We examined distal latency (DL), motor nerve conduction velocity (MCV), F-wave conduction velocity (FWCV), and sensory nerve conduction velocity (SNCV). Electric stimulation and recordings were performed with a Neuropack evoked potential/electromyogram measurement system (model MEB5508; Nihon Kohden, Co., Tokyo, Japan). For measurement of DL and MCV, the stimulation site was just behind the medial malleolus (distal) and the center of poples (proximal), and the recording site was fixed 11 cm distal to the distal stimulation site on the abductor hallucis muscle.

### Blood tests.

The following blood tests were performed in each worker: red blood cell (RBC) count, hemoglobin, hematocrit, white blood cell (WBC) count, and platelet count, using a hematocell counter (Coulter JT, Coulter Electronics, Hialeah, FL, USA), as well as fructosamine (colorimetric method), blood urea nitrogen [urease ultraviolet (UV) method], creatinine (enzyme method), total protein (Biuret method), total cholesterol (enzyme method), creatine kinase (UV *N*-acetylcysteine method), aspartate aminotransferase (UV method), alanine aminotransferase (UV method), γ-glutamyl transferase (l-γ-glutamyl-3-carboxy-4-nitroanilide substrate method), lactate dehydrogenase (Wroblewski-LaDue method), alkaline phosphatase (*p*-nitrophenol substrate method), serum creatinine (alkaline picric acid method), vitamin B_1_ (HPLC method), iron [2-nitroso-5-(*N*-propyl-*N*-sulfopropylamino)phenol method], ferritin, thyroid-stimulating hormone [radioimmunoassay (RIA)], luteinizing hormone (LH; RIA), follicle-stimulating hormone (FSH; RIA), and estradiol (RIA).

### Neurobehavioral tests and postural sway test.

Neurobehavioral testing [simple reaction time, digit span, Santa Ana, digit symbol, Benton, pursuit aiming test, Profile of Mood States (POMS)] was conducted based on the Chinese edition of the World Health Organization Neurobehavioral Core Test Battery (Chen 1988; Liang 1987) by trained Chinese researchers. Because neurobehavioral tests can be influenced by education level, we also conducted analyses with controls matched for age and education level. Postural balance was measured with a Gravicorder GS-30 stabilometer (Anima Co., Tokyo, Japan). The same instrument was used in all subjects throughout the investigation. Postural sway testing was performed as described previously (Yamamoto et al. 2001; Yokoyama et al. 1997). Briefly, the subject was asked to stand with big toes touching each other on the platform of the Gravicorder. The center of gravity was recorded every 50 msec with both eyes open for 1 min and closed for 1 min. The calculated values based on the center of gravity were *a*) the total length of excursion; *b*) envelope area; *c*) length of excursion per envelope area; *d*) rectangular area, representing the product of the range of the x-component (lateral) and that of the y-component (anteroposterior); *e*) root mean square area; *f*) the mean of *x*-axis or *y*-axis component of each recorded point; *g*) the center of range of the *x*-axis or *y*-axis component of points; *h*) power spectrum of the *x*-axis or *y*-axis at 0.02–0.2 Hz, 0.2–2.0 Hz, and 2.0–10.0 Hz, obtained by frequency analysis, with both eyes open and closed; and *i*) the Romberg quotient, representing the ratio of values measured with eyes closed to the value with eyes open for items *a* through *h*.

### Assessment of exposure to 1-BP.

Individual exposure levels during work shifts were evaluated with passive samplers (Sibata Scientific Technology Ltd., Tokyo, Japan) using the method described previously by Ichihara et al. (2004). A passive sampler was attached to each worker during one 8-hr shift and was collected immediately after the shift and kept in separate sealed bags at 4°C until analysis. The absorbed solvent in the sampler was analyzed 2 weeks after the investigation. In our previous study (Ichihara et al. 2004), we confirmed the stability of absorbed 1-BP in charcoal at 4°C for 2 weeks. For analysis, activated charcoal particles were taken from the samplers and then immersed in 2 mL carbon disulfide (Wako Pure Chemicals, Osaka, Japan) in a glass tube with a screw cap. The tube was shaken vigorously for 5 min and left to stand for 1 hr; the supernatant was then injected into a gas chromatograph equipped with an electron ionization detector (GCD system G1800A, Hewlett Packard, Palo Alto, CA, USA). The concentration of 1-BP was quantified by the selected ion mode. The detection limit was 0.007 ppm by this method. The time-weighted average (TWA) was calculated based on the formula


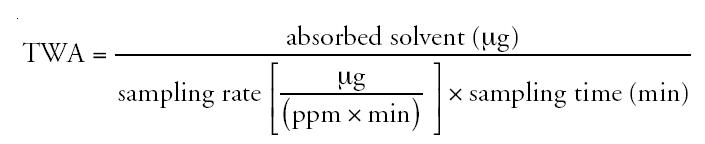


In our calculations, we used the value of 0.134 as the sampling rate of 1-BP. The value was determined by the diffusing cell method.

### Statistical analysis.

We used the paired *t*-test to compare continuous parameters of the exposure group and controls matched for age or age and education level. In this analysis, all indices of electrophysiologic studies, neurobehavioral tests, POMS test, stabilometer testing, and blood tests were compared with the age-matched controls, and the indices of neurobehavioral tests and POMS were also compared with controls matched for age and education level. We used the Wilcoxon test and Fisher’s exact test to compare the delay time and abnormality of menstrual cycles, respectively, of the exposure group and the age-matched controls. In the analysis by exposure levels, the 27 exposed workers were classified into two groups: ≤2.64 ppm (*n* = 17) and ≥ 8.84 ppm (*n* = 7); data missing (*n* = 3). For analysis by length of exposure, the 27 exposed workers were again classified into two groups: ≤ 9.31 months (*n* = 10) and ≥ 16.33 months (*n* = 16); data missing (*n* = 1). We selected these cutoff values because they divided the two peak distributions when the histograms with column width of 2.5 ppm and 5 months, respectively, were drawn, whereas no values were found between 2.64 and 8.84 ppm and 9.31 and 16.33 months. In comparisons between groups stratified with fructosamine [≤ 246 μmol/L (*n* = 14) and 248–284 μmol/L (*n* = 13)] or vitamin levels [20–30 ng/mL (*n* = 13) and ≥ 31 ng/mL (*n* = 13); data missing (*n* = 1)], the groups were divided according to the median because there was no split in the distribution that formed two peak distributions. The *t*-test was applied when comparing continuous variables (electrophysiologic tests, neurobehavioral tests, POMS test, stabilometer tests, and blood tests) by exposure levels or length of exposure as well as the levels of fructosamine or vitamin B_1_. For the analysis of delay time and frequency of menstrual cycles, we used Wilcoxon test and Fisher’s exact test, respectively, for comparison according to exposure levels, length of exposure, and the level of fructosamine or vitamin B_1_. We defined significance as the probability of *p* < 0.05.

## Results

**Figure 1:**
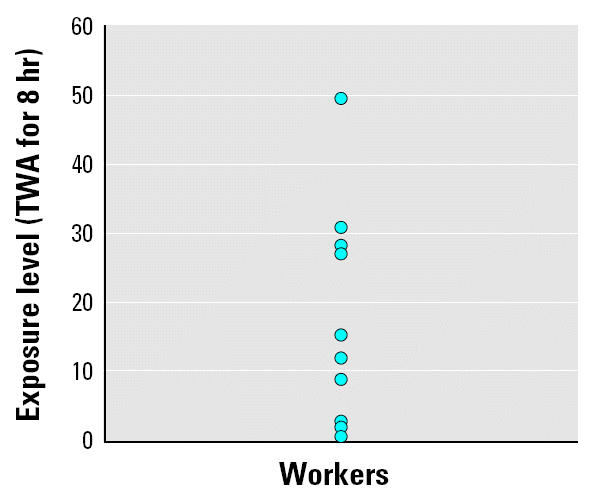
Exposure levels of each worker in a 1-BP factory (TWA for 8-hr shift). Values were obtained with passive samplers from workers who had age-matched controls (*n* = 23). Maximum = 49.19; minimum = 0.34; median = 1.61; geometric mean = 2.92 ppm.

There were no differences in age and height between 1-BP workers and the age-matched controls (Table 1). The control group had a higher education level than the exposure group. Job duration of the exposure group was shorter than for the controls, probably because the area where the workers lived had been developed quite recently, so they had engaged in agriculture before employment in the factory. Four workers in the beer factory (controls) had been exposed to various chemicals (formalin, *n* = 2; ammonia, *n* = 1; alkaline reagent, *n* = 1) in occupational settings before their present jobs. None of the workers investigated was a smoker, and only one exposed worker and one control worker were alcohol drinkers. None of the workers investigated had a history of diabetes mellitus, which could cause polyneuropathy. Individual exposure levels ranged from 0.34 to 49.2 ppm (median, 1.61 ppm; geometric mean, 2.92; Figure 1).

Bromopropane workers, all of whom were hired after 1991 (1991 workers), had significantly longer DL and lower SNCV than did the age-matched controls (Table 2). Because the main product in the factory had shifted from 2-BP to 1-BP between 1996 and May 1999 (Ichihara et al. 2004), we also analyzed data for 1999 workers to examine the effects of exposure to 1-BP only. Examination of these workers showed the only significant change to be an increase in the DL compared with age-matched controls. However, the extent of the change in any electrophysiologic parameter in the 1999 workers tended, in general, to be similar to that of the 1991 workers. Reduced vibration sensation as tested on the right toe, left toe, right finger, and left finger was detected in 15, 13, 4, and 4 female workers, respectively (Tables 3 and 4). One worker showed complete loss of vibration sense on the right toe by tuning fork stimulation. The exposure level for this worker was 1.10 ppm, and she had a relatively high DL (8.8 msec) and low MCV (43.1 m/sec), FWCV (53.7 m/sec), and SNCV (38.8 m/sec). In contrast, none of the age-matched beer workers showed any abnormalities in vibration sensation in the toe and finger. The Wilcoxon test showed significant differences in the delay time bilaterally both in the feet and in the fingers between 1991 workers and controls. Analysis of 1999 workers also showed significant prolongation of the delay time on the toes bilaterally but not in the fingers. The percentage of 1999 workers who showed reduced vibration sensation (delay time ≥ 2 sec) on both sides of the foot and in the fingers was similar to that of 1991 workers.

Neurobehavioral tests showed lower values for the forward and backward digit span, Benton visual memory test, pursuit aiming test, POMS test (scores for tension, depression, anxiety, fatigue, and confusion) in the 1-BP workers than in the controls (Tables 5 and 6). Because the education level of 1-BP workers was different from that of the age-matched controls and because the education level could affect the results of neurobehavioral tests, these tests were analyzed after matching both education level and age (Tables 5 and 6). 1-BP workers had lower levels of backward digit span; correct scores in the Benton visual memory test; completed response in the pursuit aiming test; and tension, depression, anxiety, fatigue, and confusion in the POMS test than did controls matched for age and education level. Further analysis was conducted for these neurobehavioral tests on 1999 workers (Tables 5 and 6). Significant differences with the controls were found only in the Benton visual memory test and in POMS depression and fatigue.

The postural sway tests showed significantly lower power spectrum of the *x*-axis at 2.0–1.0 Hz with eyes open and *y*-axis at 0.02–0.2 Hz with eyes closed and significantly higher power spectrum of the *y*-axis at 0.2–2.0 Hz with eyes closed (Table 7) in the 1999 workers than in the age-matched controls, but other parameters were not significantly different between the two groups (Table 7; Romberg quotients for all items, which also did not show any statistical difference, are not shown). The comparison of the 1999 workers and age-matched controls did not show any significant differences in postural sway tests (Table 7; Romberg quotients not shown).

Laboratory tests did not show any significant differences between the 1991 workers and age-matched controls (data not shown) except for significantly lower levels of vitamin B_1_ (31.0 ± 5.6 vs. 34.3 ± 5.4 ng/mL) and low WBC count (5.7 ± 1.7 × 10^3^/μL vs. 6.7 ± 1.8 × 10^3^/μL) in the 1991 workers than in age-matched controls. For 1999 workers, only the WBC count was significantly lower than in the age-matched controls. In only one worker (42 years of age) in the control group, the fructosamine level (286 μmol/L) was above the upper limit of reference value (205–285 μmol/L). This worker had rather high DL (8.24 msec) and low levels of MCV (42.5 m/sec), FWCV (49.8 m/sec), and SNCV (39.5 m/sec) but did not show abnormal vibration sensation. This worker was not included in the education-matched testing because she had no education-matched individual in the exposure group. Comparison between the two groups stratified by fructosamine levels within all exposed workers (*n* = 27) showed significant differences only in higher levels of total protein (8.22 ± 0.53 g/dL), total cholesterol (197.7 ± 32.1 mg/dL), choline esterase (ChE; 366.1 ± 86.7 IU/L), LH (14.3 ± 14.3 IU/L), WBC (6.62 ± 5.15 × 10^3^/μL), RBC (4.19 ± 0.38 × 10^6^/μL), POMS confusion (5.31 ± 4.35), and lower estradiol level (35.4 ± 25.1 pg/mL) in the high-fructosamine group compared with the low-fructosamine group (total protein, 7.64 ± 0.24 g/dL; LH, 4.2 ± 3.4 IU/L; total cholesterol, 166.5 ± 28.7 mg/dL; ChE, 288.4 ± 37.8 IU/L; WBC, 5.16 ± 0.97 × 10^3^/μL; RBC, 3.84 ± 0.38 × 10^6^/μL; POMS confusion, 2.36 ± 1.91; estradiol, 63.2 ± 38.3 pg/mL).

Fisher’s exact test did not show any difference between the 1991 and 1999 worker groups and their corresponding age-matched control groups with regard to the frequency of menstrual abnormalities after starting working in the 1-BP factory. Two workers in the exposure group had a short menstrual cycle. Similarly, one worker in the control group had a short menstrual cycle, and another reported a prolonged period of menstrual bleeding.

On the other hand, a comparison based on the exposure levels (≤ 2.64 or ≥ 8.84 ppm) showed that workers with high exposure levels showed significantly high values of MCV (56.4 ± 12.9 m/sec), FWCV (54.7 ± 2.8 m/sec), hematocrit (0.393 ± 0.032), and POMS tension (5.14 ± 1.77) and lower values of FSH (9.0 ± 6.3 mIU/mL) and POMS vigor (18.6 ± 2.5), compared with the low-exposure group (MCV, 47.3 ± 8.3 m/sec; FWCV, 52.0 ± 1.9 m/sec; hematocrit, 0.356 ± 0.034; POMS tension, 2.73 ± 1.49; FSH, 27.7 ± 35.3 mIU/mL; POMS vigor, 24.3 ± 4.0) but did not show any significant association with other examined indices. In the comparison by the length of exposure (≤ 9.31 or ≥ 16.33 months), the longer-exposure group had high levels of LH (13.5 ± 13.7 mIU/mL) and FSH (34.9 ± 34.9 mIU/mL) and lower levels of total protein (7.77 ± 0.30 g/dL) and vitamin B_1_ (29.2 ± 5.1 ng/mL) than did the shorter-exposure group (LH, 3.3 ± 1.8 mIU/mL; FSH, 5.5 ± 2.1 mIU/mL; total protein, 8.18 ± 0.66 g/dL; vitamin B_1_, 33.2 ± 5.0 ng/mL) but did not show any significant association with other examined indices.

Because the mean concentration of vitamin B_1_ was significantly lower in the exposure group than in the controls, the values were compared between the two groups stratified by vitamin B_1_ level within all exposed workers (*n* = 27). The comparison did not reveal any difference in the frequency of low vibration sensation or results of electrophysiologic tests, apart from lower levels of alkaline phosphatase (ALP; 129.3 ± 30.7 IU/L) and ChE (293.8 ± 52.8 IU/L) in the low vitamin group than high vitamin group (ALP, 169.5 ± 43.1 IU/L; ChE, 361.8 ± 84.1 IU/L).

## Discussion

In the tested factory, isopropanol, hydrogen bromide, and sulfuric acid were also used as materials in the process of producing 1-BP. These chemicals are not considered to have neurotoxic effects, so it is unlikely that the low vibration sensation or change in DL is due to these chemicals. In the last survey of the same factory (Ichihara et al. 2004), we found that the main product of this factory was shifted from 2-BP to 1-BP. 1991 Workers include the workers who were hired before May 1999 and might have been exposed to not only 1-BP but also 2-BP before 1999 (Ichihara et al. 1999). In contrast, 1999 workers were exposed to 1-BP only. Therefore, the observed changes in the DL, vibration sense in both feet bilaterally, Benton visual memory test score, and depression and fatigue in the POMS test that were noted in 1999 workers are considered to be due to exposure to 1-BP. However, the effects of 2-BP cannot be excluded in 1991 workers. The SNCV showed significant changes in the analysis of 1991 workers but not in 1999 workers with age-matched controls. This is most likely due to the lack of power as a result of the reduction in the number of subjects, given the fact that the extent of change in sensory nerve conduction, as well as other electrophysiologic parameters, and the percentage of workers who showed reduced vibration sense among 1999 workers was similar to that of 1991 workers. This explanation might also be valid for other parameters that showed significant change in 1991 workers but not in 1999 workers.

Our animal studies (Ichihara et al. 2000a; Yu et al. 1998) preceded human case reports in revealing the neurotoxicity of 1-BP, which is far more potent than that of 2-BP (Yu et al. 1999, 2001). However, the results of animal studies had certain limitations in predicting symptoms or signs in human cases; for example, animal studies cannot detect any subjective symptoms that might reflect abnormalities of sensation or the central nervous system. It is sometimes difficult especially for morphologic studies to substantiate the adverse effects on the central nervous system because the structure of the central nervous system is far more robust than that of peripheral nerves or other organs. It is also difficult to evaluate imbalance during walking in rodents because four-footed animals are completely different from bipedal humans regarding the clinical signs of imbalance. Thus, information from human cases should help us understand the toxicologic targets of 1-BP. The first case was reported by Sclar (1999), and three other cases were recently reported by our group (Ichihara et al. 2002). All four cases showed diminished vibration sensation in the toe. Moreover, the present study showed that more than half of the workers exposed to 1-BP suffered from reduced vibration sensation. Considered together, these results suggest that vibration sensation in the toe might be susceptible to exposure with 1-BP. The previously reported cases also complained of urinary incontinence; numbness in the perineum, low back, and front of the thighs or buttocks; or headache (Ichihara et al. 2002); however, our factory workers did not report any such symptoms. This difference might depend on the levels or period of exposure to 1-BP because it is possible that our workers adapted to low levels but longer periods of exposure, leading to unawareness of symptoms.

In comparisons with age-matched controls, both the 1991 workers and 1999 workers showed prolonged DL but no change in MCV. This prolongation of DL without decrease in MCV parallels the results of animal studies, which showed earlier changes in DL than MCV in the tail nerve (Ichihara et al. 2000a). Such a pattern of changes might indicate predominant deterioration of the distal portion of the peripheral nerve or delay in chemical transmission between nerve terminals and muscle.

Comparison of data of 1991 workers with age-matched controls showed that the exposure group had lower levels of forward and backward digit span, Benton scores, pursuit aiming test scores, and POMS tension, depression, anxiety, fatigue, and confusion than did the controls. Because education level could influence the results of neurobehavioral tests, the results of the tests were reanalyzed after matching age and education levels. This reanalysis also revealed changes in the above items excluding forward digit span. When the analysis was limited to the 1999 workers, significant differences were found only in Benton visual memory test scores, POMS depression, and POMS fatigue, which could reflect the lack of power due to the small sample number. Digit span, pursuit aiming test, and the POMS test are considered the most sensitive indicators of exposure to organic solvents or neurotoxic agents such as lead (Zhou et al. 2002). Poorer performance in the POMS test was also observed in a Venezuelan study of workers exposed to organic solvents (Escalona et al. 1995). The present results of neurobehavioral tests could also suggest that 1-BP adversely affects the central nervous system in humans. Postural sway tests showed higher power of the *y*-axis (anterior–posterior sway) at 0.2–2.0 Hz and lower at 0.02–0.2 Hz with eyes closed, although such significant differences were not observed in 1999 workers. These results might be important because the cases found in the United States also showed unstable balance in walking. Clinically, patients with cerebellar disease and anterior lobe atrophy show anteroposterior sway, often with a spontaneous high-frequency body tremor of around 3 Hz (Diener et al. 1984). This anteroposterior sway might resemble the present result of the increase in the power of the *y*-axis at 0.2–2 Hz. However, the results of the postural sway tests noted in our study await further confirmation because the presence of cerebellar disorder in the formerly reported cases or present workers is not conclusive, and it is possible to attribute the unstable balance to a disorder of the peripheral nerves or spinal cord.

Diabetes mellitus could be a common confounding factor related to neurologic disorders by solvent intoxication. HbA1C and fructosamine are used as long-term (Bunn et al. 1976) and intermediate-term (1–3 weeks) (Baker et al. 1983) indicators of glucose levels in clinical settings. In the present study, we measured serum fructosamine levels. For the measurement of HbA1C, the blood samples had to be kept at 4°C but not frozen. However, the long transportation from the factory site to the laboratory could have potentially caused hemolysis of the collected blood and thus may have resulted in marked variability and errors in estimations. For this reason, HbA1C was not measured in the present study. The comparison between the exposed group and the controls did not show any difference in the level of fructosamine, and the comparison between the high-fructosamine group and low-fructosamine group within the exposed group also did not show any difference in indices related to the nervous system.

The levels of vitamin B_1_ were lower in the entire exposure group than in the controls and in the longer-exposure group compared with the shorter-exposure group. Lack of vitamin B_1_ is known to cause polyneuropathy, but the relatively low level of vitamin B_1_ in the 1-BP factory workers could not fully explain the neurologic abnormalities. First, the level of vitamin B_1_ in the exposed workers ranged from 20 to 43 ng/mL, which was within the normal range (20–50 ng/mL). Second, the low-level vitamin B_1_ group showed no neurologic deficit such as vibration sensation or electrophysiologic indices, apart from a low score of POMS confusion, which would be weak evidence in substantiating the adverse effects on the nervous system.

Letz and Gerr (1994a, 1994b) investigated the confounding factors that could affect nerve conduction velocity and amplitude as well as vibrotactile and thermal thresholds, based on data from 4,464 subjects. Their studies revealed that the major covariates were height, examiner, skin temperature, and body mass index for sural sensory nerve and height, examiner, age, and body mass index for peroneal motor nerve conduction velocities. For vibrotactile threshold in toe, the major covariates were height, examiner, age, and body mass index. Our study design could control for the effect of examiner-, sex-, and age-matching pairs but not skin temperature-, body height-, or body mass index-matching pairs. Although body height was comparable on average between the exposure group and the controls and workers were acclimated to the room temperature before the electrophysiologic studies, the lack of pair matching for height, skin temperature, and body mass index should be carefully noted as a limitation of this study. Previous animal experiments demonstrated that exposure to 1-BP disrupted the estrous cycle and inhibited follicular development (Yamada et al. 2003). Two patients who worked in a cushion company in the United States also reported temporary irregularities of menstrual cycle (Ichihara et al. 2002). Although the exposure level for the two patients was not evaluated directly, such levels would be higher than 60–261 ppm, which were determined with the third case from the same factory after the former two cases were identified and ventilation was improved in the workplace. On the other hand, our study did not demonstrate significant differences in the prevalence of menstrual cycle abnormalities between the two groups. This might be due to the difference in exposure levels between U.S. cases (≥ 60–261 ppm) and our Chinese 1-BP factory workers (0.34–49.19 ppm).

Comparisons based on the exposure period showed higher levels of FSH and LH in the longer-exposure group than in shorter-exposure group. One explanation for this difference is that our group included four elderly women, who were excluded from the paired *t*-test analysis because of the lack of matched controls and who had high levels of FSH (42–100 mIU/mL) and LH (16–42 mIU/mL). Analysis based on exposure level did not show any relationship between exposure levels and these parameters, which were different between the exposure group and age-matched controls (paired *t*-test). The present analysis by exposure period and level has certain limitations. First, the number of subjects was too small and did not control for age. Second, the experimental design allowed only a single measurement of the exposure level, although the task of workers was not fixed and thus the exposure levels could vary. The exposure levels in 1999 in the same factory ranged from 0.9 to 170.5 ppm (geometric mean = 52.5 ppm) (Ichihara et al. 2004), which was far higher than in the present study. It is possible that the workers were exposed to 1-BP at higher levels than those measured in our study. Further assessment of long-term exposure levels is required to determine the relationship between 1-BP and exposure levels.

In summary, the present study suggested that exposure to 1-BP produces adverse effects on peripheral sensory and motor nerves and/or the central nervous system in humans. Estimation of long-term exposure levels is required to confirm the precise association between the health effects of 1-BP and exposure levels.

## Correction

In the manuscript published online, the numbers of workers listed in Table 1, especially in the footnotes, were incorrect; also, the statistical significance of values for the right and left fingers for 1999 workers and age-matched controls was incorrect. These errors have been corrected here.

## Figures and Tables

**Table 1:** Characteristics of workers.

Characteristic	1-BP exposed (*n* = 23)	Control (*n* = 23)
Age (years)	36.2 ± 5.7*^a^*	36.2 ± 5.2
Height (cm)	160.3 ± 6.6*^a^*	158.8 ± 5.9
Education		
Elementary school	4	4
Junior high school	19	12
High school	0	6
University	0	1
Job duration (months)	27 ± 31	168 ± 67
Past job exposure to chemicals	0	4*^b^*
Previous medical condition	2*^c^*	8*^d^*

Data for age, height, and job duration are mean ± SD.

Other values are numbers of workers.

a Not significantly different from the controls (paired *t-*test).

b Includes formalin (2), ammonia (1), alkaline (1).

c Includes cholecystitis (1), contraceptive use (1).

d Includes anemia (2), gastritis (2), hysteromyoma (2), oophoritic cyst (1), cholecystitis (1), taking antihypertensive medications (1).

**Table 2:** Electrophysiologic indices of workers exposed to 1-BP and of the controls.

	1991 workers	Age-matched controls for 1991	1999 workers	Age-matched controls for 1999
No. of pairs	23		12	
DL of nervus tibialis (msec)	8.05 ± 2.17*	5.96 ± 1.38	8.36 ± 2.38*	6.06 ± 1.43
MCV of nervus tibialis (m/sec)	49.8 ± 10.3	49.9 ± 8.2	51.3 ± 12.0	51.7 ± 10.7
FWCV of nervus tibialis (m/sec)	52.8 ± 3.5	55.1 ± 3.2	51.8 ± 2.8	55.0 ± 2.9
SNCV of nervus suralis (m/sec)	39.2 ± 3.5*	46.2 ± 6.6	39.2 ± 2.6	47.5 ± 8.5

Data are mean ± SD.

* *p* < 0.05 compared with age-matched controls (paired *t*-test).

**Table 3:** Number of workers with reduced vibration sensation in the foot.

	1991 workers and age-matched controls (*n* = 23 pairs)				1999 workers and age-matched controls (*n* = 12 pairs)			
	Right foot*		Left foot*		Right foot*		Left foot*	
Delay time*^a^* (sec)	1-BP workers	Controls	1-BP workers	Controls	1-BP workers	Controls	1-BP workers	Controls
< 2	8	23	10	23	5	12	5	12
2	0	0	1	0	0	0	1	0
3	3	0	1	0	1	0	1	0
4	2	0	4	0	1	0	1	0
5	2	0	1	0	1	0	0	0
6	4	0	4	0	3	0	2	0
8	3	0	1	0	1	0	1	0
10	0	0	1	0	0	0	1	0
∞*^b^*	1	0	0	0	0	0	0	0

a Delay time for vibration sensation by tuning fork stimulation (see “Materials and Methods” for details); time 0 is the time when the worker reported becoming unaware of the vibration.

b One worker felt no vibration sense in the right foot.

* *p* < 0.05, Wilcoxon test.

**Table 4:** Number of workers with reduced vibration sensation in the finger.

	1991 workers and age-matched controls (*n* = 23 pairs)				1999 workers and age-matched controls (*n* = 12 pairs)			
	Right finger*		Left finger*		Right finger		Left finger	
Delay time*^a^* (sec)	1-BP workers	Controls	1-BP workers	Controls	1-BP workers	Controls	1-BP workers	Controls
< 2	19	23	19	23	10	12	10	12
2	3	0	2	0	2	0	2	0
3	1	0	2	0	1	0	0	0

a Delay time for vibration sensation by tuning fork stimulation (see “Materials and Methods” for details); time 0 is the time when the worker reported becoming unaware of the vibration.

* *p* < 0.05, Wilcoxon test.

**Table 5:** Results of neurobehavioral tests in the 1-BP group and controls matched for age or for age and education (mean ± SD).

Test	1991 workers (age-matched controls)	1991 workers (age/education-matched controls)	(age/education-matched controls) 1999 workers
No. (pairs)	22	12	6
Simple reaction time (sec)	0.38 ± 0.12 (0.36 ± 0.12)	0.38 ± 0.12 (0.36 ± 0.12)	0.40 ± 0.14 (0.39 ± 0.12)
Digit span (digits recalled) forward	10.6 ± 2.3* (11.7 ± 1.4)	10.8 ± 2.5 (11.8 ± 1.3)	10.2 ± 3.1 (12.0 ± 1.1)
Digit span (digits recalled) backward	4.5 ± 2.2* (5.8 ± 1.8)	5.0 ± 2.6* (5.6 ± 1.4)	4.2 ± 2.3 (6.2 ± 1.6)
Santa Ana preferred hand	35.2 ± 3.6 (36.6 ± 4.8)	35.3 ± 4.0 (36.1 ± 4.3)	36.0 ± 2.4 (35.8 ± 5.2)
Santa Ana nonpreferred hand	33.5 ± 4.6 (32.8 ± 5.1)	33.8 ± 5.2 (33.7 ± 5.6)	32.8 ± 4.4 (35.5 ± 5.9)
Digit symbol (no. completed)	47.0 ± 17.5 (54.0 ± 10.2)	48.6 ± 19.8 (55.5 ± 5.6)	45.3 ± 21.9 (56.7 ± 6.7)
Benton (no. correct)	7.2 ± 1.7* (8.3 ± 1.4)	7.8 ± 1.5* (8.2 ± 1.3)	7.3 ± 1.8* (8.3 ± 1.0)
Pursuit aiming test (no. completed)	103.1 ± 16.9* (119.9 ± 19.1)	101.6 ± 17.9* (119.3 ± 20.4)	98.0 ± 11.4 (125.7 ± 17.0)

* *p* < 0.05, paired *t*-test.

**Table 6:** Results of POMS tests in the 1-BP group and controls matched for age or for age and education (mean ± SD).

Test	1991 workers (age-matched controls)	1991 workers (age/education-matched controls)	1999 workers (age/education-matched controls)
No. (pairs)	20	12	6
Profile of mood state			
Tension	4.4 ± 3.9* (7.7 ± 7.1)	4.1 ± 5.2* (10.2 ± 8.5)	6.8 ± 7.0 (9.6 ± 7.2)
Depression	4.8 ± 7.5* (10.5 ± 13.0)	5.6 ± 10.1* (13.3 ± 17.1)	10.0 ± 14.4* (12.8 ± 16.0)
Anxiety	4.1 ± 5.0* (10.2 ± 10.2)	4.7 ± 6.3* (12.6 ± 13.2)	7.0 ± 9.0 (13.4 ± 12.7)
Vigor	22.2 ± 4.3 (20.7 ± 6.7)	23.7 ± 3.9 (20.9 ± 6.9)	23.4 ± 4.5 (21.4 ± 9.2)
Fatigue	3.1 ± 2.6* (6.4 ± 4.0)	3.0 ± 3.4* (6.7 ± 5.2)	4.4 ± 4.8* (7.2 ± 3.8)
Confusion	3.7 ± 3.7* (7.1 ± 4.3)	3.3 ± 4.4* (7.7 ± 5.6)	5.0 ± 6.2 (5.6 ± 5.0)

* *p* < 0.05, paired *t*-test.

**Table 7:** Stabilometer test results of 1-BP exposure group and controls.

	1991 Workers	Age-matched controls	1999 Workers	Age-matched controls
No. (pairs)	23		12	
LNG (cm)				
Eyes open	71.7 ± 15.5	69.9 ± 20.8	71.5 ± 19.3	74.0 ± 19.4
Eyes closed	100.4 ± 25.1	91.1 ± 27.3	106.3 ± 29.7	95.0 ± 26.0
E AREA (cm^2^)				
Eyes open	3.38 ± 1.26	3.69 ± 2.86	3.60 ± 1.52	3.88 ± 2.64
Eyes closed	4.94 ± 2.27	4.56 ± 3.62	5.65 ± 2.67	4.80 ± 4.15
LNG E AREA (per cm)				
Eyes open	22.9 ± 6.3	24.9 ± 10.9	21.8 ± 6.6	26.3 ± 13.7
Eyes closed	23.1 ± 7.9	26.3 ± 10.4	22.0 ± 8.8	28.8 ± 12.2
REC AREA (cm^2^)				
Eyes open	7.53 ± 2.76	8.26 ± 6.39	7.87 ± 3.42	8.51 ± 6.08
Eyes closed	10.5 ± 5.6	10.3 ± 8.6	12.8 ± 6.1	10.5 ± 9.6
RMS (cm^2^)				
Eyes open	1.62 ± 0.70	1.98 ± 1.67	1.82 ± 0.91	2.09 ± 1.46
Eyes closed	2.05 ± 1.01	2.05 ± 1.65	2.30 ± 1.25	2.22 ± 2.01
Mx (cm)				
Eyes open	0.019 ± 0.581	−0.123 ± 1.162	−0.166 ± 0.485	−0.008 ± 0.724
Eyes closed	0.010 ± 0.573	0.053 ± 1.228	−0.044 ± 0.557	0.217 ± 0.679
My (cm)				
Eyes open	−2.43 ± 1.15	−2.09 ± 1.37	−2.70 ± 1.05	−2.20 ± 1.41
Eyes closed	−2.29 ± 1.06	−2.06 ± 1.28	−2.50 ± 0.95	−2.41 ± 1.07
XO (cm)				
Eyes open	−0.004 ± 0.621	−0.142 ± 1.170	−0.186 ± 0.627	−0.009 ± 0.756
Eyes closed	0.119 ± 0.657	−0.003 ± 1.347	0.106 ± 0.771	0.231 ± 0.788
YO (cm)				
Eyes open	−2.50 ± 1.15	−2.07 ± 1.39	−2.79 ± 1.14	−2.22 ± 1.41
Eyes closed	−2.29 ± 1.01	−2.30 ± 1.42	−2.48 ± 0.86	−2.48 ± 1.09
Power spectrum of *x*-axis (lateral)				
Eyes open (%)				
0.02–0.2 Hz	61.1 ± 12.4	54.7 ± 17.0	62.5 ± 12.1	53.7 ± 14.5
0.2–2.0 Hz	38.5 ± 12.3	42.1 ± 13.6	37.1 ± 11.9	45.8 ± 14.4
2.0–10 Hz	0.36 ± 0.21*	0.46 ± 0.21	0.38 ± 0.24	0.46 ± 0.21
Eyes closed (%)				
0.02–0.2 Hz	45.7 ± 17.3	47.9 ± 12.2	48.5 ± 17.8	46.1 ± 12.7
0.2–2.0 Hz	52.5 ± 19.7	49.2 ± 16.3	48.4 ± 21.8	49.2 ± 19.7
2.0–10 Hz	0.53 ± 0.37	0.59 ± 0.33	0.60 ± 0.44	0.58 ± 0.34
Power spectrum of *y*-axis (anteroposterior)				
Eyes open (%)				
0.02–0.2 Hz	66.6 ± 14.0	70.7 ± 11.4	73.5 ± 11.1	70.9 ± 10.1
0.2–2.0 Hz	32.4 ± 12.8	28.9 ± 11.4	26.1 ± 11.1	28.6 ± 10.1
2.0–10 Hz	0.97 ± 2.62	0.42 ± 0.28	0.35 ± 0.16	0.45 ± 0.35
Eyes closed (%)				
0.02–0.2 Hz	51.6 ± 14.0*	61.2 ± 13.7	54.0 ± 11.6	55.0 ± 11.2
0.2–2.0 Hz	47.3 ± 13.0*	38.3 ± 13.7	45.4 ± 11.6	44.4 ± 11.2
2.0–10 Hz	1.03 ± 2.38	0.50 ± 0.28	0.56 ± 0.26	0.57 ± 0.25

Abbreviations: E, envelope; LNG, length of excursion; Mx, mean of *x*-axis (lateral) component of each recorded points; My, mean of *y*-axis (anteroposterior) component of each recorded points; REC AREA, rectangular area; RMS, root mean square area; XO, center of range of *x*-axis component of points; YO, center of range of *y*-axis component of points. Data are mean ± SD.

* *p* < 0.05, paired *t*-test. No significant difference was found between the exposed group and the controls in the Romberg quotient for all items (the ratio of values measured with eyes closed to the values with eyes open; data not shown).

## References

[b1-ehp0112-001319] Baker JR, O’Connor JP, Metcalf PA, Lawson MR, Johnson RN (1983). Clinical usefulness of estimation of serum fructosamine concentration as a screening test for diabetes mellitus. Br Med J.

[b2-ehp0112-001319] Bunn HF, Haney DN, Kamin S, Gabbay KH, Gallop PM (1976). The biosynthesis of human hemoglobin A1c. Slow glycosylation of hemoglobin in vivo. J Clin Invest.

[b3-ehp0112-001319] Chen Z (1988). WHO neurobehavioral test indicators and evaluation standard. Chin J Prev Med.

[b4-ehp0112-001319] Diener HC, Dichgans J, Bacher M, Gompf B (1984). Quantification of postural sway in normals and patients with cerebellar diseases. Electroencephalogr Clin Neurophysiol.

[b5-ehp0112-001319] Environ Tech 2001. Response to NTP-CERHR Expert Panel Draft Report on Reproductive and Developmental Toxicity of 1-Bromopropane. Melrose Park, IL:Environ Tech International, Inc.

[b6-ehp0112-001319] Escalona E, Yanes L, Feo O, Maizlish N (1995). Neurobehavioral evaluation of Venezuelan workers exposed to organic solvent mixtures. Am J Ind Med.

[b7-ehp0112-001319] Ichihara G In press. Neuro-reproductive toxicities of 1-bromopropane and 2-bromopropane. Int Arch Occup Environ Health. 10.1007/s00420-004-0547-9 15812677

[b8-ehp0112-001319] Ichihara G, Ding X, Yu X, Wu X, Kamijima M, Peng S (1999). Occupational health survey on workers exposed to 2-bromopropane at low concentrations. Am J Ind Med.

[b9-ehp0112-001319] Ichihara G, Kitoh J, Yu X, Asaeda N, Iwai H, Kumazawa T (2000a). 1-Bromopropane, an alternative to ozone layer depleting solvents, is dose-dependently neurotoxic to rats in long-term inhalation exposure. Toxicol Sci.

[b10-ehp0112-001319] Ichihara G, Li W, Ding X, Peng S, Yu X, Shibata E (2004). A survey on exposure level, health status, and biomarkers in workers exposed to 1-bromopropane. Am J Ind Med.

[b11-ehp0112-001319] Ichihara G, Miller JK, Ziolkowska A, Itohara S, Takeuchi Y (2002). Neurological disorders in three workers exposed to 1-bromopropane. J Occup Health.

[b12-ehp0112-001319] Ichihara G, Yu X, Kitoh J, Asaeda N, Kumazawa T, Iwai H (2000b). Reproductive toxicity of 1-bromopropane, a newly introduced alternative to ozone layer depleting solvents, in male rats. Toxicol Sci.

[b13-ehp0112-001319] Letz R, Gerr F (1994a). Covariates of human peripheral nerve function: I. Nerve conduction velocity and amplitude. Neurotoxicol Teratol.

[b14-ehp0112-001319] Letz R, Gerr F (1994b). Covariates of human peripheral nerve function: II. Vibrotactile and thermal thresholds. Neurotoxicol Teratol.

[b15-ehp0112-001319] Liang Y (1987). An introduction to WHO neurobehavioral core test battery. J Ind Occup Dis.

[b16-ehp0112-001319] Sclar G (1999). Encephalomyeloradiculoneuropathy following exposure to an industrial solvent. Clin Neurol Neurosurg.

[b17-ehp0112-001319] Wang H, Ichihara G, Ito H, Kato K, Kitoh J, Yamada T (2002). Biochemical changes in the central nervous system of rats exposed to 1-bromopropane for seven days. Toxicol Sci.

[b18-ehp0112-001319] Wang H, Ichihara G, Ito H, Kato K, Kitoh J, Yamada T (2003). Dose-dependent biochemical changes in rat central nervous system after 12-week exposure to 1-bromopropane. Neurotoxicology.

[b19-ehp0112-001319] World Medical Association 2002. World Medical Association Declaration of Helsinki: Ethical Principals for Medical Research Involving Human Subjects. Available: http://www.wma.net/e/policy/pdf/17c.pdf [accessed 20 July 2004].

[b20-ehp0112-001319] Yamada T, Ichihara G, Wang H, Yu X, Maeda K, Tsukamura H (2003). Exposure to 1-bromopropane causes ovarian dysfunction in rats. Toxicol Sci.

[b21-ehp0112-001319] Yamamoto R, Kinoshita T, Momoki T, Arai T, Okamura A, Hirao K (2001). Postural sway and diabetic peripheral neuropathy. Diabetes Res Clin Pract.

[b22-ehp0112-001319] Yokoyama K, Araki S, Murata K, Morita Y, Katsuno N, Tanigawa T (1997). Subclinical vestibulo-cerebellar, anterior cerebellar lobe and spinocerebellar effects in lead workers in relation to concurrent and past exposure. Neurotoxicology.

[b23-ehp0112-001319] Yu X, Ichihara G, Kitoh J, Xie Z, Shibata E, Kamijima M (1999). Effect of inhalation exposure to 2-bromopropane on the nervous system in rats. Toxicology.

[b24-ehp0112-001319] Yu X, Ichihara G, Kitoh J, Xie Z, Shibata E, Kamijima M (1998). Preliminary report on the neurotoxicity of 1-bromopropane an alternative solvent for chlorofluorocarbons. J Occup Health.

[b25-ehp0112-001319] Yu X, Ichihara G, Kitoh J, Xie Z, Shibata E, Kamijima M (2001). Neurotoxicity of 2-bromopropane and 1-bromopropane, alternative solvents for chlorofluorocarbons. Environ Res.

[b26-ehp0112-001319] Zhou W, Liang Y, Christiani DC (2002). Utility of the WHO neurobehavioral core test battery in Chinese workers—a meta-analysis. Environ Res.

